# Demographic predictors of emotional intelligence among radiation therapists

**DOI:** 10.1002/jmrs.277

**Published:** 2018-04-23

**Authors:** Trakis Stami, Fernandez Ritin, Parrish Dominique

**Affiliations:** ^1^ St George Cancer Care Centre Kogarah NSW Australia; ^2^ St George Hospital, Level 1, James Laws House University Of Wollongong Wollongong NSW Australia; ^3^ Faculty of Science, Medicine and Health (SMAH) University Of Wollongong Wollongong NSW Australia

**Keywords:** Emotional intelligence, predictors, radiation therapists, survey

## Abstract

**Introduction:**

Contemporary health care services are more productive and successful when their health professionals have emotional intelligence (EI). The objective of this study was to explore the demographic predictors of EI among radiation therapists working in cancer care centres in NSW, Australia.

**Methods:**

Data were collected using a cross‐sectional self‐administered survey. Emotional intelligence was measured using the Trait Emotional Intelligence Questionnaire‐ Short version (TEIQue – SF). Multiple regression analysis was used to identify if age, years of experience, gender, highest level of education obtained or level of current employment were predictors of EI.

**Results:**

A total of 205 radiation therapists participated in this study. The mean scores for Global EI, emotionality, self‐control, wellbeing and sociability dimensions were 5.16 (SD = 0.6), 5.3 (SD = 0.7), 4.9 (SD = 0.9), 5.7 (SD = 0.8) and 4.7 (SD = 0.8) respectively. Age and level of current employment were identified as predictors of global EI. Gender and level of education were significant predictors of the EI emotionality dimension. Levels of employment along with level of education were both significant predictors of the sociability dimension of EI.

**Conclusions:**

Being a young radiation therapist, female, and having higher levels of employment and higher levels of education were predictors of EI. Given that level of education and level of employment are both amendable demographic factors, strategies to address these factors to reduce the effects of emotional struggle experienced by radiation therapists in their work need to be implemented.

## Introduction

Emotional intelligence (EI) has been defined as a blend of personal and interpersonal competencies that affect one's behaviour, thinking and interactions with others.^1^ Salovey and Mayer^2^ first coined emotional intelligence defining it as “the ability to monitor one's own and others’ feelings and emotions, to discriminate among them and to use this information to guide one's thinking and actions” (p. 189). This definition and the conceptualisation of emotional intelligence has been further developed through research and practice.^3^ While there are a number of alternate models of emotional intelligence such as the Bar‐On,^4^ and Salovey and Mayer^2^ models, the Goleman^5^ model is widely used.

The Goleman model of EI organises emotional intelligence into four dimensions; self‐awareness and self‐management and social awareness and social skills.^5^ These four dimensions of EI each comprise a suite of personal or interpersonal competencies that are essential for health care professionals in order to provide optimal patient care as well as work synergistically as part of a multidisciplinary team.^6^ Theorists have viewed EI as a trait rather than a cognitive ability.^7^, ^8^, ^9^ According to them trait EI is related to people's personality and is formed by their emotional self‐perceptions and emotional traits. Unlike ability, trait EI involves behavioural characteristics and self‐perceived capabilities and is measured through self‐report.^7^, ^8^, ^9^ Therefore, for this study it was decided that trait EI would be used as a measurement of EI among radiation therapists (RT).

### Benefits of EI

Various benefits of EI relating to performance, stress, job satisfaction, burnout and patient care have been reported among health care professionals. Evidence from the literature suggests that people with higher EI are better able to achieve goals, maintain strong relationships and have greater performance in social relations.^10^ Emotional intelligence related to self‐management, social awareness and social skills has been found to be highly relevant and important requirement for leadership.^3^

An inverse correlation was reported between EI and stress among nurses working in private and public hospitals,^11^ with those nurses who had higher EI being less stressed. A study in the Netherlands, of nurses working with people with mental illness and severe behavioural problems found that low EI was associated with higher burnout in female nurses. In an observational study, undertaken on 110 medical doctors higher self‐rated EI has been significantly associated with less burnout (*P* < 0.001) and higher job satisfaction (*P* < 0.001).^12^ An integrative literature review of 39 empirical research articles that focused on EI in nursing found positive impact of EI on leaders thus influencing employee retention, quality of patient care and patient outcomes. This investigation concluded that EI should be explicitly taught within nursing education.^13^ A descriptive, correlational study involving 135 nurses from three hospitals in counties of New York, USA identified a positive correlation between nurses’ self‐compassion and EI.^14^

### The association between EI and demographic factors

The influence of demographic factors on the EI of individuals has been explored in studies and literature. A number of studies have identified a positive correlation between EI and age with older people reporting higher emotional intelligence.^15^, ^16^ An American study involving 405 participants aged between 22 and 70, found that EI increased slightly with age.^17^ In this study linear regression analysis was conducted in which age was the independent variable and EI was the dependent variable. The authors suggest that emotional intelligence develops cumulatively as a consequence of life experiences. While these findings are logical, findings in more recent literature suggests that age is not a predictor of EI.^18^ This is a premise that will be investigated in this research study.

There are studies that have reported women to be more socially skilful compared to men.^8^ An Australian study exploring the work stress and EI of mental health nurses found that female nurses with less experience in mental health had lower EI. This was not the case however in the male participants.^19^ High EI among women has been attributed to biological and social factors. The biological factors include the larger size of the brain area, which processes emotions, in women compared to men.^20^ The social factors are related to the innate or learnt behaviours of men and women, where women are taught and encouraged to be more empathetic and men are conditioned to be more constructive.^20^ Furthermore, researchers have found that higher levels of emotional intelligence in women may be due to the influences and nurturing roles between the mother and her child in which the male children are likely to obtain less emotional expression from their mothers than female children.^21^

Educational level has been identified as another demographic factor that influences EI. In a study undertaken on 212 professionals working in a mental health setting there was a statistically significant correlation between EI and educational levels, with those who had higher levels of education demonstrating greater EI.^22^

### Studies of EI and RTs

A comprehensive search of the literature identified five publications investigating EI among radiographers of which three were undertaken among radiography students.^23^, ^24^, ^25^ The remaining two studies investigated EI among qualified diagnostic and therapy radiographers (RT).^26^, ^27^ In the study by Mackay^26^ the mean global EI score for radiographers was 5.27 (SD = 0.691) and in the second study the mean global EI scores ranged from 5.14 to 5.60.^27^ The study by Mackay 2013 also indicated that there was no statistically significant difference in EI levels between diagnostic and therapy radiographers. This result could be related to the unequal sample size of the two groups where the number of therapy radiographers comprised of only up to 18% of the total sample.

In Australia, there is a significant difference in the role of diagnostic and therapy radiographers in oncology. The main role of the diagnostic radiographer is to deliver high‐quality medical imaging to enable medical specialists in making accurate informed diagnosis of the patient's illness. On the other hand RTs are responsible for the “design, accurate calculation and delivery of a prescribed radiation dose over a course of treatment to the patient.”^28^ In addition to having scientific and technological knowledge, the role of the RTs also involves counselling patients to allay their fears and anxieties about their diagnosis and treatment.^28^

In many instances RTs provide radiation therapy for patients over a period of 4–8 weeks. Hence, patients undergoing radiation treatment develop a rapport with their RT who also provides them and their families with emotional comfort. Illness and prolonged treatment regime can have an impact not only on the patient but also on the RT. Therefore, RTs are required to have empathy and compassion and acknowledge patients’ vulnerability, while at the same time being capable of managing their own emotions in a professional manner.

While there is extensive literature published on EI among people in executive positions, across a range of professions and among students, there is a paucity of data relating to the emotional intelligence among qualified RTs. Therefore, the aim of this study was to investigate the EI levels and the demographic predictors of EI among qualified RTs.

## Methods

### Research design

This study adopted a quantitative, non‐experimental, cross‐sectional research design.

### Sample

All RTs who met the criteria for professional entry to radiation therapy as per the Medical Radiation Practice Board of Australia (MRPBA) guidelines^29^ and irrespective of their level of employment and working in any of the 15 public cancer care centre's in NSW were eligible to participate in this study.

Currently in NSW RTs are employed between levels 1–6, where level 1 is classified as professional development year and level 6 classified as chief RTs.^29^ Radiation therapists who were on leave were excluded from this study. In addition, those who were undertaking their professional development year or a supervised practice programme were also excluded.

### Data collection instrument

Data for this study were collected through a self‐administered survey. The data collected included demographic information, as well as measures of EI traits. The demographic details collected included, gender, age, educational level, level of employment and years of experience as a RT. Emotional intelligence was measured using the Trait Emotional Intelligence Questionnaire‐Short Form (TEIQue‐SF). The TEIQue‐SF is a 30‐item self‐report measure that comprises four dimensions namely Wellbeing (6 items), Self‐control (6 items), Emotionality (8 items) and Sociability (6 items).^30^ The remaining four items contributed only to the measure of Global EI, which was measured by aggregating the scores for all 30 items. Wellbeing as used in this instrument refers to a generalised sense of wellbeing extending from past achievements to future expectations, accompanied by high self‐esteem, and includes the facets of self‐esteem, trait happiness and trait optimism. The Emotionality dimension reflects the ability to identify and express feelings, and to use these faculties to maintain close relationships with significant others, and it includes the facets of emotion perception, emotion expression, trait empathy and relationships. The Sociability dimension, regarding the capacity to assert oneself as well as to influence others’ emotions and decisions, includes the facets of social awareness, emotion management and assertiveness. The Self‐Control dimension, concerning the ability to regulate one's impulses and emotions, as well as managing external pressures and stress, includes the facets of emotion regulation, stress management and impulsiveness.^31^

The TEIQue‐SF has been shown to have high reliability and validity with Cronbach's alpha ranging from 0.65 to 0.85.^30^, ^32^, ^33^, ^34^ This tool requires participants to rate their degree of agreement with each item on a seven‐point Likert‐type scale with responses ranging from completely disagree (1) to completely agree (7).

### Data collection method

Prior to commencement of this research, approval was sought from the chief RT at each of the 15 cancer care centres in NSW. This was done by providing a 10 minute presentation about the study via a teleconference at the chief RT meeting. All chief RTs agreed to participate and nominated the RT educators at their centres as the point of contact. Two weeks later the RT educators at each of the participating cancer care centres were provided a detailed account of the study rationale, design, participant recruitment and data collection tools. The RT educators were also provided with a presentation as well as a copy of the study proposal to provide information to RTs in their centre, about the study, during a regular in‐service session. Educators informed the researcher of the number of RTs working at their centre so that an appropriate number of surveys could be prepared for each therapist. One week later an agreed number of individual research packs, consisting of the invitation letter, informed consent sheet, questionnaires and a return envelope addressed to the primary researcher, were delivered by mail to the educators for distribution at their centre. In order to minimise coercion RTs were informed that participating in the study was voluntary and non‐participation would have no effect on their employment. Educators were requested to return by mail, all surveys, regardless of whether they were completed or not, at the end of 8 weeks. Consent was assumed by the completion and return of the surveys. In an attempt to increase the response rate, the educators were sent follow up reminders every 2 weeks. Ethics approval to conduct the study across NSW was obtained from the South Eastern Sydney Local Health District Human Research Ethics Committee (HREC ref no: 15/049 LNR/15/POWH/180) and the University of Wollongong Human Research Ethics Committee (2017/449).

### Data analysis

The data collected for this study were entered into survey monkey and exported into the Statistical Package for the Social Sciences V21 (SPSS) for data analysis. Data were cleaned and reviewed for any missing values. Missing data were replaced according to author guidelines.^35^ To maintain integrity of the data set, 10% of the data were audited by a person not associated with the project.

Categorical data were presented as percentages and continuous data werepresented as means and standard deviation (SD). TEIQue items were reversed according to guidelines.^30^ A Global Trait EI score was calculated by averaging the scores for all 30 items. Similarly, scores for the 4 dimensions were calculated by averaging all of the items associated with the dimensions.^30^ All demographic variables were included in a standard multiple linear regression analysis to determine the predictors of overall EI and the predictors of each EI dimension. Prior to conducting the analysis, the demographical variables of age, current employment as a RT and highest level of qualification that had more than one category were transformed into categorical variables with only two categories and coded as 0 and 1 to undertake the regression analysis. Age was combined into two categories: ≤39 and ≥40. Current employment as a RT was dichotomised at level 2 or level 3.1 and greater. Highest level of qualification was dichotomised at bachelor's degree and lower or master's degree and higher. The chief Beta (*B*) values and the 95% confidence intervals were calculated in the multiple regression analyses. Statistical significance was set at *P* less than 0.05.

## Results

### Sample description

During the period of the survey in July 2015, there were 300 RTs working in the 15 cancer care centres in NSW. Completed questionnaires were received from 205 RTs yielding an overall response rate of 68%. Respondents in this study were predominantly female between 20 and 39 years of age (33% 20–29% and 33% 30–39%) and currently employed as an RT at level 2 (53%). The years of experience as a RT following the professional development year ranged from 6 months to 40 years with the mean being 12 years (SD = 9.2). The demographic characteristics of respondents are presented in Table 1.

**Table 1:** Demographic characteristics of the participants (*n* = 205)

	Frequency (%)
Gender^a^	
Female	157 (71.7)
Male	45 (20.5)
What age group do you belong to?	
20–29	72 (32.9)
30–39	73 (33.3)
40–49	38 (17.4)
50–59	21 (9.6)
60–69	1 (0.5)
What is the level of your current employment as a RT?^a^	
Level 2	117 (53.4)
Level 3.1	15 (6.8)
Level 3.2	11 (5.0)
Level 4.1	34 (15.5)
Level 4.2	19 (8.7)
Level 5	6 (2.7)
Level 6	2 (0.9)
Are you currently undertaking any postgraduate or higher degree courses?	
Yes	18 (8.2)

a Missing data.

### Emotional intelligence

The mean global EI for participants was 5.16 (SD = 0.6) (range 2.7–6.9). The mean scores for the EI dimensions were 5.3 (SD = 0.7) (range 3.0–7.0) for the emotionality dimension, the self‐control dimension was 4.8 (SD = 0.8) (range 2.3–7.0), the wellbeing dimension was 5.7 (SD = 0.8) (range 3.17–7.0) and the sociability dimension was 4.7 (SD = 0.8) (range 2.17–7.0)**.**

### Emotional dimension

Radiation therapists aged between 20 and 39 years had higher means scores compared to those aged between 40 and 69 years (mean difference = 0.11, 95% CI = −0.11, 0.33). Those with a higher level of employment (level 3.1–level 6) had higher emotional scores compared to those with a lower level of current employment (mean difference = 0.02, 95% CI = −0.23, 0.17). Female RTs had significantly higher emotional scores compared to their male counterparts (mean difference = 0.27, 95% CI = −0.52, −0.03) (Table 2).

**Table 2:** EI scores

Variable	Emotional	Self‐control	Well‐being	Sociability	Global
Gender					
Male	5.10 (SD = 0.72)	4.88 (SD = 0.76)	5.60 (SD = 0.85)	4.68 (SD = 0.85)	5.04 (SD = 0.60)
Female	5.38 (SD = 0.72)	4.85 (SD = 0.89)	5.79 (SD = 0.71)	4.74 (SD = 0.83)	5.20 (SD = 0.60)
Age					
20–39	5.36 (SD = 0.72)	4.91 (SD = 0.86)	5.78 (SD = 0.72)	4.78 (SD = 0.82)	5.21 (SD = 0.59)
40–69	5.24 (SD = 0.73)	4.75 (SD = 0.86)	5.69 (SD = 0.81)	4.62 (SD = 0.85)	5.05 (SD = 0.63)
Current employment					
Level 2	5.31 (SD = 0.76)	4.80 (SD = 0.90)	5.79 (SD = 0.76)	4.63 (SD = 0.86)	5.14 (SD = 0.64)
Level 3.1–6	5.34 (SD = 0.67)	4.93 (SD = 0.80)	5.69 (SD = 0.73)	4.86 (SD = 0.77)	5.19 (SD = 0.54)
Level of Education					
Bachelors and lower	5.26 (SD = 0.71)	4.87 (SD = 0.86)	5.73 (SD = 0.75)	4.67 (SD = 0.84)	5.13 (SD = 0.60)
Postgraduate	5.57 (SD = 0.77)	4.78 (SD = 0.84)	5.82 (SD = 0.74)	5.03 (SD = 0.73)	5.28 (SD = 0.57)

### Self‐control dimension

Radiation therapists aged between 20 and 39 years had higher means scores compared to those aged between 40 and 69 years (mean difference = 0.15, 95% CI = −0.10, 0.42). Those with a higher level of employment (level 3.1–level 6) had higher self‐control scores compared to those with a lower level of current employment (mean difference = 0.13, 95% CI = −0.37, 0.11). Male RTs had higher self‐control scores compared to their female counterparts (mean difference = 0.03, 95% CI = −0.25, 0.33) (Table 2).

### Well‐being dimension

Radiation therapists aged between 20 and 39 years had higher means scores compared to those aged between 40 and 69 years (mean difference = 0.09, 95% CI = −0.13, 0.32). Those with a lower level of employment (Level 2) had higher well‐being scores compared to those with a higher level of current employment (level 3.1–level 6) (mean difference = 0.09, 95% CI = −0.12, 0.30). Female RTs had higher well‐being scores compared to their male counterparts (mean difference = 0.19, 95% CI = −0.44, 0.06) (Table 2).

### Sociability dimension

Radiation therapists aged between 20 and 39 years had higher means scores compared to those aged between 40 and 69 years (mean difference = 0.15, 95% CI = −0.09, 0.40). Those with a higher level of employment (level 3.1–level 6) had higher sociability scores compared to those with a lower level of current employment (mean difference = 0.23, 95% CI = −0.46, 0.00). Female RTs had higher sociability scores compared to their male counterparts (mean difference = 0.06, 95% CI = −0.36, 0.04) (Table 2).

### Global EI

Radiation therapists aged between 20 and 39 years had higher means scores compared to those aged between 40 and 69 years (mean difference = 0.16, 95% CI = −0.02, 0.34). Those with a higher level of employment (level 3.1–level 6) had higher global scores compared to those with a lower level of current employment (mean difference = 0.05, CI = −0.22, 0.16). Female RTs had higher global scores compared to their male counterparts (mean difference = 0.16, 95% CI = −0.52, −0.03) (Table 2).

### Predictors of EI

Separate standard multiple regression analyses were performed for the following dependant variables: global emotional intelligence, emotionality, self‐control, wellbeing and sociability. The demographic characteristics included as predictor variables were age, years of experience, gender, highest level of education obtained and level of current employment.

### Predictors of global EI

The multiple regression model to predict global emotional intelligence among RTs was significant and accounted for 6.1% of the variance, *R* ^2^_Adj_ = 0.037 *F* (5,189) = 2.475, *P* = 0.034. The only significant predictor of global EI was age with RT's aged between 20 and 39 years having higher global EI (*B* = −0.341; 95% CI −0.65, −0.03; *P* = 0.031) (Table 3).

**Table 3:** Predictors of EI

Model	Unstandardised coefficients	Sig.	95% Confidence interval for *B*	
	*B*		Lower bound	Upper bound
Global EI				
Constant	4.99	0.000	4.78	5.21
Age	−0.341	0.031	−0.650	−0.032
Level of current employment	0.136	0.205	−0.075	0.346
Gender	0.176	0.091	−0.029	0.381
Years of experience	0.003	0.701	−0.013	0.020
Highest level of education	0.143	0.213	−0.083	0.368
Emotional				
Constant	5.090	0.000	4.838	5.341
Age	−0.215	0.255	−0.586	0.156
Level of current employment	0.126	0.325	−0.126	0.379
Gender^a^	0.273	0.030	0.027	0.519
Years of experience	−0.003	0.779	−0.023	0.017
Highest level of education	0.311	0.025	0.040	0.581
Sociability				
Constant	4.566	0.000	4.283	4.849
Age	−0.417	0.051	−0.835	0.001
Level of current employment	0.329	0.024	0.044	0.615
Gender^a^	0.065	0.647	−0.213	0.342
Years of experience	0.003	0.822	−0.020	0.025
Highest level of education	0.374	0.017	0.069	0.679
Self‐Control				
Constant	4.83	0.000	4.529	5.138
Age	−0.439	0.055	−0.889	0.010
Level of current employment	0.225	0.148	−0.081	0.532
Gender^a^	−0.022	0.885	−0.320	0.276
Years of experience	0.007	0.576	−0.017	0.031
Highest level of education	−0.111	0.506	−0.439	0.217
Well‐being				
Constant	5.57	0.000	5.31	5.84
Age	−0.245	0.219	−0.638	0.147
Level of current employment	−0.123	0.365	−0.391	0.145
Gender	−0.191	0.150	−0.070	0.451
Years of experience	0.011	0.327	−0.011	0.032
Highest level of education	0.099	0.495	−0.187	0.386

a Negative coefficients indicate higher scores for females.

### Predictors of the four EI dimensions

The multiple regression model to predict emotionality was significant and accounted for 6.8% of the variance, *R* ^2^_Adj_ = 0.044 *F* (5,189) = 2.77, *P* = 0.019. Female gender (*B* = 0.273; 95% CI 0.027, 0.519; *P* = 0.030) and having postgraduate qualifications (*B* = 0.311; 95% CI 0.040, 0.581; *P* = 0.025) was associated with higher emotionality dimension of EI.

The multiple regression model to predict sociability was significant and accounted for 7.9% of the variance, *R* ^2^_Adj_ = 0.054 *F* (5,189) = 3.223, *P* = 0.008. Having postgraduate qualifications (*B* = 0.374; 95% CI 0.069, 0.679; *P* = 0.017) and high level of current employment (*B* = 0.329; 95% CI 0.044, 0.615; *P* = 0.024) were significant and independently associated with the sociability dimension (Table 3). None of the demographic variables were significant predictors of the self‐control and well‐being dimensions.

## Discussion

The results from this study demonstrates that the Global EI as well as the wellbeing, self‐control, emotionality and sociability dimensions of RTs is higher than that reported in the literature among first year student radiographers^24^ and the normative data,^27^ but was lower than qualified radiographers.^24^ In contrast the emotionality dimension was higher than that of radiographers.^24^ This could be due to the fact that RT has a greater contact with the patients compared to radiographers and hence have developed the ability to control their emotions.

The results of this study indicated that younger RTs had higher global EI. It has been reported that the older a person becomes the more likely they are to have a positive outlook, less neuroticism and better emotional control.^36^ In addition, they become more aware of the fragility and complexities of life, which enables them to better handle their emotions.^36^ However, the findings from this study did not conclude this result, in fact it found the complete opposite; that the younger RTs had higher emotional intelligence. One inference for this result could be that RTs are exposed on a regular basis to traumatic and distressing situations, where their patient and families are grappling with the grief of a potentially terminal disease. Exposure to these traumatic and distressing situations could have contributed to the development of EI among younger RTs.^37^ Thus, instead of becoming more emotionally intelligent as they get older, the time that RTs have the greatest EI would be when they are younger which is synonymous with the findings in this study.

What is interesting in this study is that although younger RTs had higher EI, years of experience were not found to be a predictor of EI. This result is in contrast to studies that have found a positive correlation between years of experience and EI.^38^ A possible explanation for this dissonance could be that radiotherapy departments are constantly evolving, due to an increase in new techniques and technologies. Associated with this evolution is the need for practitioners to keep abreast of learning and development that is involved with all these changes. This evolution adds to the constant pressures faced by RTs who also deal with high patient loads and maintaining focus on providing a service that keeps up with increasing work demands. The prevalence of these demands and conditions negates the benefits and advantages of years of experience including increased EI, which explains the result of years of experience not being a predictor of EI in this study.

Being female was identified as a predictor of the emotional dimension of EI, which is a result consistent with the findings of other published EI studies.^8^, ^39^ Emotionality relates to being able to identify and express emotions as well as maintain intimate relationships with others. The finding in this study, that being female is a predictor of the emotionality dimension of EI may not be related to being an RT. Rather, the explanation for this finding could simply be associated with the fact that females possess learnt behaviours, resultant from nurturing, which make them innately more attune with their feelings and capable in sustaining relationships.

Previous literature has acknowledged that an increase in the level of education improves EI.^22^ This study did not replicate these findings, with higher levels of education not being a predictor of Global EI. This result may be explained by the fact that the majority of RTs only gain the level of education needed for their professional role. Once they are in the field, many RTs will not seek further education unless it is required to develop technical competence. Thus the finding, that higher levels of education are not a predictor of Global EI is logical because the skills associated with Global EI are not related to technical competence. In addition, studies that demonstrated an association between EI and education levels were undertaken in general population where there could have been a variation in education levels across individuals which might have influenced the results. This study was undertaken in RTs where the range of education level was constrained hence the association between education and EI may have not manifested.

However, higher levels of education were found to be a predictor of higher emotionality which is a subscale of the TEIQue. The Emotionality dimension reflects the ability to identify and express feelings, and to use these faculties to maintain close relationships with significant others, and it includes the facets of emotion perception, emotion expression, trait empathy, and relationships. Participants could have acquired these skills when undertaking higher education programmes such as management and leadership as most of these programmes offer subjects, courses or workshops relating to emotional intelligence. Obtaining these skills has a direct impact on the RTs’ ability to engage with others and express their feelings.

The Sociability dimension, regarding the capacity to assert oneself as well as to influence others’ emotions and decisions, includes the facets of social awareness, emotion management, and assertiveness. This study found that the EI sociability dimension was greater among those with both a high level of employment and a high level of education. Explanations for this result could be due to the fact that confidence and experience, both of which it is reasonable to assume are gained as a consequence of higher levels of employment and education, are going to enhance ones’ sociability. Furthermore, the RT's environment of teamwork and close affiliations within the multidisciplinary team require the skills of sociability, so these will logically be increased as a result of their regular and ongoing employment in more senior roles.

In this study none of the demographic variables were significant predictors of the self‐control and well‐being dimensions of EI. A possible reason for these results could be that radiotherapy environments are highly technological environments dealing with high patient loads and thus managing external pressures and stress is more synonymous with self‐management than any of the demographic predictors tested in this study. Similarly, wellbeing is not a major focus of RT environments that are more concerned about providing a service rather than ensuring the happiness and optimism of RTs.

The major strength of this study was that it included a broad cohort of RTs who worked in cancer care centres across NSW. In addition, the study was conducted in a rigorous manner using validated instruments. A high response rate of 68% of the population sample is also strength of the study and makes the findings both meaningful and generalisable. Despite the evidence, some limitations inherent in undertaking in such a study need to be acknowledged. First, this study used a self‐selected sample whom may have been highly motivated. Another limitation of the study was that the R‐squared values were quite low for each model which suggests that each model only explained a small amount of variation in EI. Other measured factors could likely explain the inter‐individual variation in EI. Emotional Intelligence is a complex variable hence further research needs to be undertaken looking at reasons why older RT have lower levels of global EI. In addition, it would be interesting to replicate this study in other professions that are emotionally demanding such as police, doctors and lawyers.

## Conclusion

Radiation therapy is an emotionally demanding profession and focuses mainly on practical skills. This study has contributed new and valuable insights about EI among RTs. Global EI was significantly associated with younger age. Level of employment was also a significant predictor of global EI as well as the sociability factor of EI. Level of education was a significant predictor of the sociability and emotional dimension of EI. The results should, however, be treated with caution, because EI is a highly complex phenomenon that is influenced by numerous social and cultural factors and not merely demographic characteristics. Furthermore large scale trials are warranted to establish a causal relationship between education level, employment level and EI.

## Conflict of Interest

The authors declare no conflict of interest.
